# The chloroplast genome elucidates the origin of mulberry in Central Asia

**DOI:** 10.3389/fpls.2025.1592308

**Published:** 2025-08-28

**Authors:** Caihui Wang, Yutian Yang, Lu Yang, Xue Ling, Zhikun Ma, Tongqian Zou, Jian Ma, Ming Zhang

**Affiliations:** ^1^ School of Cultural Heritage, Northwest University, Xi’an, China; ^2^ China-Central Asia “the Belt and Road” Joint Laboratory on Human and Environment Research, School of Cultural Heritage, Northwest University, Xi’an, China; ^3^ Key Laboratory of Cultural Heritage Research and Conservation, Northwest University, Xi’an, China; ^4^ Silk Road’ University of Tourism and Cultural Heritage, Samarkand, Uzbekistan

**Keywords:** *Morus* spp., population diversity, Central Asia, chloroplast genome, Silk Road

## Abstract

The mulberry (*Morus* spp.), an economically important crop along the ancient Silk Road, is widely distributed in Central Asia, yet its origins and dispersal history in the region remain unclear. Chloroplast genomes are extensively used for species identification, evolutionary analyses, and phylogenetics. We resequenced and *de novo* assembled 25 chloroplast genomes from Samarkand, Uzbekistan. Our results reveal that they predominantly comprise two species: white mulberry (*Morus alba*), originating from East Asia, particularly China; and black mulberry (*Morus nigra*), native to the Caucasus and Western Asia. Strong genetic links to both eastern and western source populations indicate that bidirectional Silk Road exchanges shaped the region’s mulberry population. These findings provide new insights into the geographic distribution and dispersal history of mulberries. This study enhances our understanding of the ecological and historical dynamics that shaped the spread of economically significant plants.

## 1 Introduction

Cultural exchange across the Eurasian continent may have commenced in the prehistoric era, possibly as early as the fifth millennium. This interaction subsequently intensified during the Bronze Age, paving the way for the establishment of the ancient Silk Road under the Han Dynasty (approximately 2,200 years ago) (Dong et al., 2017; Long, 2024). During this period, crops domesticated in Southwest Asia and northern China were introduced to Central Asia and northwest China (Jones et al., 2011; Filipović et al., 2020; Richards et al., 2022; Wang et al., 2022; Zhao et al., 2023). During the Eastern Han Dynasty (~25–220 CE), the historical Silk Road became a transcontinental network that fostered political, economic, and cultural exchanges across Eurasia further facilitated the dissemination of crops (Barisitz, 2017; Shan et al., 2024).

Sericulture boosted silk production and trade. Mulberry (*Morus* spp.) as the sole food source for silkworms (*Bombyx mori*), underpins the development of the silk industry, and serves as an economically significant crop along the ancient Silk Road (Dai et al., 2023). The genus *Morus* belongs to the Moraceae family and includes approximately 10–24 species with diverse morphological variations, found throughout all temperate areas and the mountain regions of tropical Africa, Indonesia, and South America (Wu et al., 2003; Xia, 2015; Rohela et al., 2020). Mulberry cultivation dates back over 5,000 years, leading to the development of numerous varieties and cultivars that have since spread to multiple areas (Zeng et al., 2022). The historical exchanges of germplasms during mulberry domestication and cultivation may have facilitated frequent inter- and intraspecific introgression events (Dai et al., 2023). Nowadays, the most widely cultivated species are *Morus alba* (white mulberry) and *Morus nigra* (black mulberry), both of which are economically important for their roles in sericulture as well as for their nutritional and medicinal properties. Differences in leaf area, morphology, and petiole length between these species potentially reflect adaptations to distinct environments (Yang et al., 2023; Guncheva, 2024). *M*. *alba*, native to China, is widely distributed across northern and southern China and was disseminated to Europe and the Americas via the Silk Road as early as 1st century (Wang et al., 2022; Dai et al., 2023). In contrast, *M*. *nigra* originated from the Caucasus region of West Asia and was introduced into southern Xinjiang, China during the 16th century (Zeng et al., 2013).

For Centuries, Central Asia has served as a vital hub, connecting Europe to East Asia and acting as a bridge for transferring goods, technology, and ideas (Barisitz, 2017). The heart of the Silk Road leads through Uzbekistan, which became one of the first region in Central Asia to cultivate mulberry for sericulture (Gu and Pang, 1999). Today, Uzbekistan is home to over 40,000 hectares of mulberry fields, comprising nearly 300 million trees, predominantly of *M*. *alba* and *M*. *nigra* (Gu and Pang, 1999; Mir-Makhamad et al., 2023). Research on mulberry in Central Asia has gained increasing attention from archaeologists as it can provide valuable insights into trade and cultural exchanges along the Silk Road. Historical and genetic studies have suggested that mulberries were introduced to Central Asia via human migration and trade along the Silk Road (Yin, 1998; Zhou and Chen, 2017; Dai et al., 2023; Nasirillaev et al., 2023). Although historical accounts and genetic studies provide valuable insights into the spread of mulberry along the Silk Road, the lack of samples from Central Asia has limited the ability to trace its genetic origins in this region directly.

Chloroplast genomes are used extensively for species identification, evolutionary analyses, and phylogenetics studies due to their high sequence conservation associated with maternal inheritance, nonrecombinant nature, and a conserved gene structure (Zhang et al., 2023). In this study, we collected fresh leaf samples from 25 old mulberry trees in Samarkand, Uzbekistan, performed whole-genome resequencing, and assembled their chloroplast genomes *de novo*. By integrating these data with 119 mulberry chloroplast genomes from Asia, Europe, and the Americas, we aim to elucidate the mulberries’ genetic origins and dispersal patterns of the mulberries in Central Asia.

## 2 Materials and methods

### 2.1 Sample collection and sequencing

For the purpose of whole genome resequencing, fresh leaves were collected from 25 mulberry trees in Samarkand, Uzbekistan (39°39′N, 66°58′E). Given that mulberry trees in the study area are predominantly distributed along roadsides, we employed a purposive, street-based random sampling. We intentionally selected trees that appeared morphologically old (avoiding newly planted ones, as they may have been transplanted recently and thus could not accurately reflect the historical genetic background of the local population). To ensure broad spatial representation and minimize selection bias, specimens were obtained along a circular route of about 5 km in circumference radiating from the “Silk Road” University of Tourism and Cultural Heritage, with intervals of approximately 100–500 meters.

To efficiently disrupt plant cell walls and membranes, we prepared a fresh 2% CTAB extraction buffer and autoclaved it before use. The composition of the CTAB extraction buffer (for 100 mL) was as follows: 2% (w/v) CTAB, 5% (w/v) NaCl, 20 mM Tris-HCl (pH 8.0), 4 mM EDTA (pH 8.0), and 0.2% (w/v) PVP-360. Due to the significant reduction of protein contamination by *β*-mercaptoethanol, we added 2% (v/v) *β*-mercaptoethanol to the buffer immediately before use and then preheated it to 65°C. We ground plant tissue samples to a fine powder in liquid nitrogen in a grinding tube and added preheated extraction buffer to the powder, followed by further grinding to obtain a homogeneous mixture. And then we incubated the lysate at 65°C for 60 minutes with agitation (400-1,400 rpm). After incubation, we cooled samples to room temperature and centrifuged them at 12,000 × g for 5 minutes at ambient temperature. To remove proteins and other cellular debris, we sequentially extracted the supernatant with phenol:chloroform:isoamyl alcohol (25:24:1, v/v), followed by chloroform:isoamyl alcohol (24:1, v/v). After each addition and thorough mixing, we centrifuged the mixture at 12,000 × g for 10 minutes at room temperature. Given the efficacy of 75% ethanol in removing residual salts and the stabilizing properties of TE buffer for DNA storage, we washed DNA with 75% ethanol and dissolved it in TE buffer for storage. We constructed genomic DNA libraries with insert sizes of 300–500 bp using the BGI Optimal DNA Library Prep Kit. Library amplification was carried out for 6 PCR cycles (98°C for 10s, 60°C for 30s, and 72°C for 30s per cycle), as recommended for 200 ng of input DNA. The final libraries were sequenced on the DNBSEQ-G400 platform at BGI (Beijing Genomics Institute) to generate 150 bp paired-end reads. We conducted all procedures, including DNA extraction, library construction, and resequencing, by BGI. For comparative analysis, we *de novo* assembled chloroplast genomes from our whole-genome data and downloaded published *Morus* chloroplast genomes from various regions via GenBank (https://www.ncbi.nlm.nih.gov/genbank/) (**Supplementary Table 1**).

### 2.2 Quality control of whole-genome resequencing data

Raw sequencing reads were processed by SOAPnuke software (Chen et al., 2018) using the parameters ‘-n 0.001 -l 10 -q 0.5 –adaMR 0.25 –polyX 50 –minReadLen 150’. Reads were discarded if they met any of the following criteria: containing ≥25.0% adapter sequence alignment (allowing up to 2 base pair mismatches), reads length shorter than 150 bp, comprising ≥0.1% ‘N’ bases, containing polyX sequences longer than 50 bp, or having ≥50.0% of bases with Phred quality scores below 10 (Q < 10). Clean reads were output using the Phred+33 quality encoding system (enabled by the -G parameter).

### 2.3 *De novo* assembly of the chloroplast genome

In order to construct a comparative chloroplast genome dataset, chloroplast genomes were *de novo* assembled from genome wide data in this study and public databases based on the settings (-R 48 -k 21,45,65,85,105) of GetOrganelle (v1.7.7.0) (Jin et al., 2019). To avoid potential reference bias from confounding the phylogenetic resolution of *M*. *alba* and *M*. *nigra*, we employed the genetic distant *M*. *notabilis* (GenBank Accession Number: PQ083379) as a neutral seed for the assembly. It is noteworthy that assemblies that could not be circularized, were manually adjusted in Bandage (Wick et al., 2015). Considering that this step may lead to the inversion of the inverted repeat (IR) regions and the single copy (SC) regions (Baiakhmetov et al., 2021), Gepard (v2.1.0) (Krumsiek et al., 2007) was employed to identify the typical circular quadripartite architecture of the chloroplast genome. As long as a different architecture was identified from the reference genome (GenBank: PQ083379), adjustments were made with a Python script (https://github.com/Xwb7533/chloroplast_analysis/blob/main/02_IR.py, 03_LSC_adj.py, 04_SSC_adj.py).

### 2.4 Principal component analysis

To visually illustrate the genetic relationship of Samarkand samples, we performed principal component analysis (PCA) using R packages on a dataset of 96 samples. The dataset included 71 samples of *M*. *alba*, *M*. *alba* var. *multicaulis*, and *M*. *nigra*, alongside the 25 Samarkand samples. Firstly, data (‘*.fasta*’ file) was read using the *Biostrings* package. Then, the *adegenet* package was utilized to call the single nucleotide polymorphisms (SNPs) by converting the *.fasta* data into a binary format, where 0 represents the reference allele and 1 represents the alternative allele (Zhang et al., 2022a). Finally, PCA analysis was executed with the *vegan* package. The analysis results were visualized using the *ggplot2* package.

### 2.5 Network construction

A median-joining network analysis was performed to investigate the genetic structure using the same dataset (n = 96) as the PCA analysis. Data from FASTA format were converted to NEXUS format using an R script, and the resulting ‘*.nexus*’ file was loaded as the input file into PopART (v1.7) (Leigh and Bryant, 2015) to construct and visualize a median-joining network. Furthermore, we modified the color, font, and other attributes in the edit menu. In the figure, short horizontal lines represent the number of base substitutions, while small black dots represent possible haplotypes predicted by the software.

### 2.6 Phylogenetic analysis

**Figure 1:**
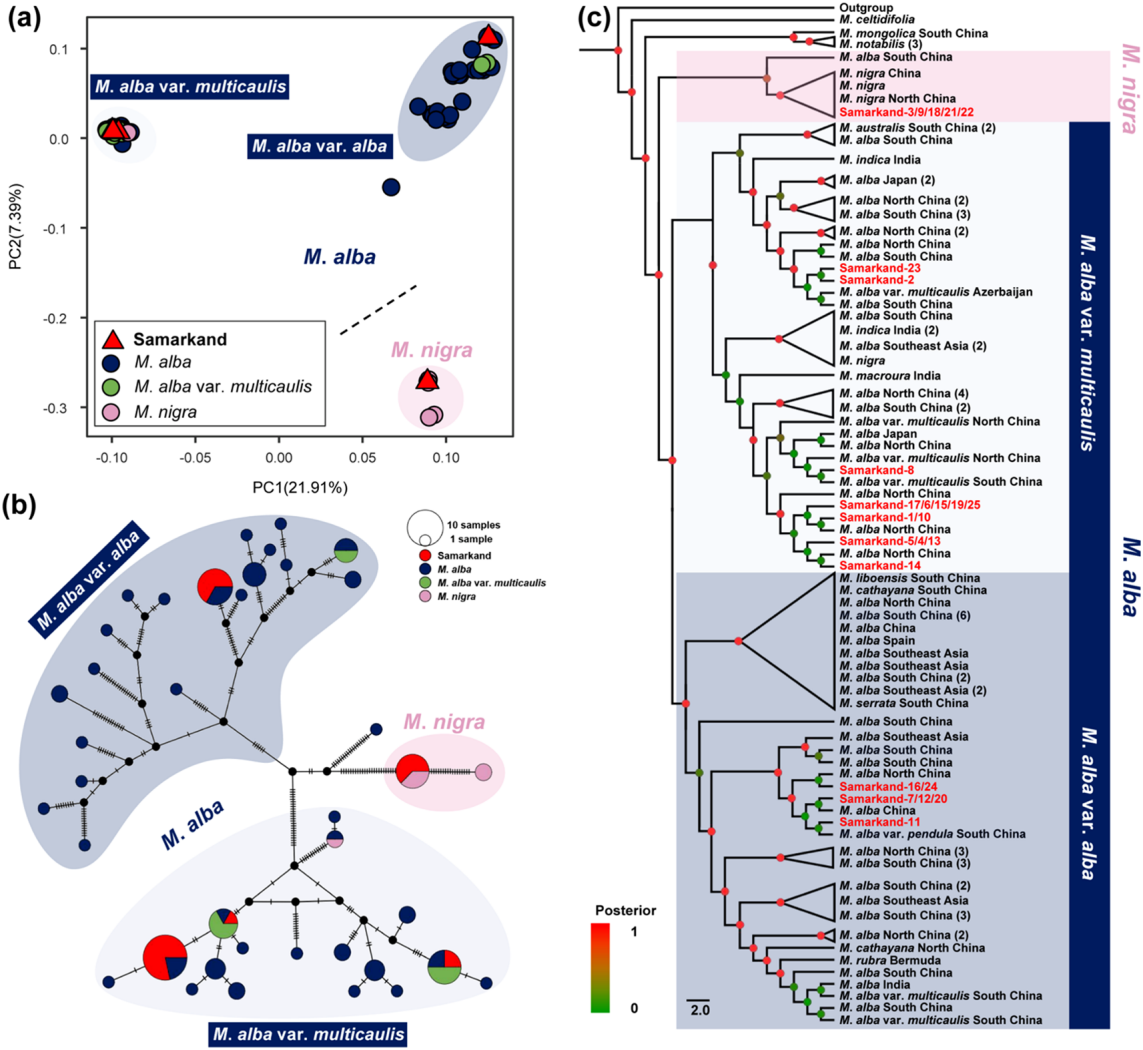
Genetic analysis of the mulberry population in Samarkand, Uzbekistan. **(a)** Principal component analysis. The dotted line distinguishes *M. alba* and *M. nigra* group. *M. alba* group were initially divided into.*M*. *alba* var. *alba* and *M*. *alba* var. *multicaulis.* Different colors represent different species. **(b)** Median-joining network. The hash marks on the lines connecting the circles indicate the number of mutations separating the haplotypes. Different colors represent different species. **(c)** Bayesian phylogenetic tree among *Morus* species based on their chloroplast genomes with 119 *Morus* samples and three outgroups (*Ficus*, *Broussonetia*, and *Morus*). Posterior values are marked by dots ranging from red to green, reflecting support values from 1 to 0. Samples from Samarkand are shown in red.

To assess the evolutionary relationships of Samarkand samples, we constructed phylogenetic trees based on chloroplast genomes using both maximum likelihood and Bayesian methods, which are sufficiently sensitive to capture the subtle genetic differentiation between closely related species. Phylogenetic analysis was carried out using the comparative chloroplast genome panel, with sample names updated following the latest taxonomic classification from iPlant (https://www.iplant.cn/). We selected the chloroplast genome sequences of *Broussonetia papyrifera* (GenBank: MZ662865), *Ficus carica* (GenBank: KY635880), and *Morus mesozygia* (GenBank: MZ274134) as outgroups following Zeng et al. (2022). The sequences were aligned using MAFFT (v7.520) (Katoh and Standley, 2013), and the alignments were trimmed using trimAl (v1.4.rev15) (Capella-Gutiérrez et al., 2009) with automated1 parameter, a heuristic setting that identifies optimal trimming thresholds based on gap and similarity scores. Then, the best-fit models of nucleotide substitution were determined based on jModelTest (v2.1.10) (Darriba et al., 2012). Subsequently, using RAxML (v8.2.12) (Kozlov et al., 2019), we constructed a maximum likelihood tree with the following parameters: -m GTRGAMMAI -N 1000. At the same time, a Bayesian tree was estimated with Beast (v1.10.4) (Suchard et al., 2018), applying a “GTR” substitution model, an uncorrelated relaxed clock with a lognormal distribution, and a “Speciation Birth-Death Process” for the tree prior, chain length of 800 million, and sampling frequency of 5000. After discarding 10% of the trees as burn-in (Zhang et al., 2022b), the remaining trees were used to generate the MCC tree, which estimated posterior probabilities. All estimated parameters were reviewed in Tracer (v1.7) (Rambaut et al., 2018), ensuring that a reasonable effective sample size (ESS) was greater than 200, which indicated a sufficient posterior distribution quality. Finally, the phylogenetic trees were visualized in Figtree (v1.4.4). To reduce computational burden, we filtered the data by retaining only one representative sequence from each set of identical sequences, resulting in a Bayesian phylogenetic tree constructed from a dataset containing 119 *Morus* chloroplast genomes and three outgroups (*Ficus*, *Broussonetia*, and *Morus*) (**Figure 1**; **Supplementary Table 1**).

### 2.7 Pairwise distances computation

Based on the *p*-distance model and utilizing the same dataset used for phylogenetic analysis, we computed pairwise distances among the comparative chloroplast genome panel using MEGA (v11.0.3) (Kumar et al., 2018) with the default settings and 1,000 bootstraps. The boxplot was visualized using the *ggplot2* package in R.

### 2.8 Nucleotide diversity and selective pressure analysis

For the calculation of genetic diversity indices and selective pressure, the “DNA Polymorphism” and “Synonymous and NonSynomymous Substitutions” functions of DnaSP (v6.12.03) (Rozas et al., 2017) were used, based on the same dataset employed for phylogenetic analysis. To ensure a valid comparison, we restricted our analysis to regions with more than five samples, focusing exclusively on white mulberry and black mulberry. Regarding black mulberry, two regions were considered: China and Uzbekistan. For white mulberry, thirteen regions were established: China_all, China_Chongqing, China_Guangdong&Guangxi, China_Hainan, China_Heilongjiang&Liaoning&Hebei&Beijing, China_Hunan&Hubei, China_Shandong&Anhui, China_Shanxi&Shaanxi, China_Xinjiang, China_Yunnan, Uzbekistan, Southeast Asia (including Vietnam and Thailand), and India. As for samples from China, we initially grouped these samples into a single population for comparison with those from Uzbekistan. Considering the large geographical differences, we further divided the Chinese samples into nine populations based on their geographical regions. Regions with limited sample sizes but contiguous locations were merged into single populations.

## 3 Results

### 3.1 Sample information

We conducted whole-genome resequencing on fresh leaf samples from 25 old mulberry trees in Samarkand, Uzbekistan, generating 54.77-60.22 million clean reads per sample. Most reads were high quality: 98.23-99.00% of the reads had a minimum q-score of Q20, 94.01-96.22% of the reads reached Q30; and the GC content was uniform (34.06-34.63% across all samples). From this dataset, we successfully assembled 25 Samarkand chloroplast genomes. The missing data ratio for all Samarkand samples was low, ranging from 5.32% to 5.41% (**Supplementary Table 1**).

**Figure 2:**
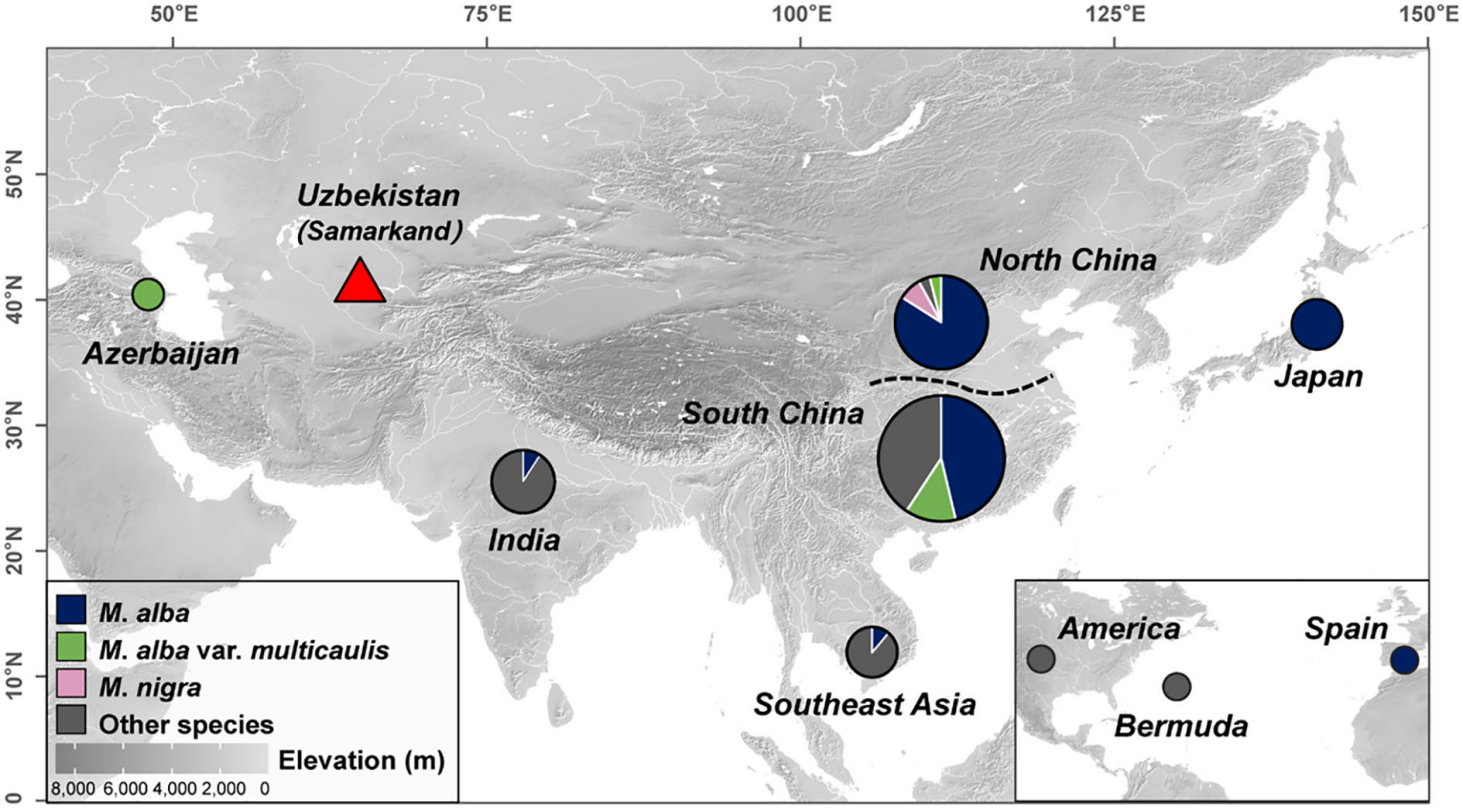
Geographic distribution of samples. Geographical locations of the selected *Morus* samples. The pie size represents the number of samples, from small to large representing 1, 2-10, 11-20, 20-30, and greater than 30. Different colors are used to distinguish different species (“Other species” comprises all *Morus* species mentioned in **Supplementary Table 1**, except for *M*. *alba*, *M*. *alba* var. *multicaulis*, and *M*. *nigra*.). Elevation is represented by a gray color ramp. The darker the color, the higher the elevation; the lighter the color, the lower the elevation. The white areas represent the water area.

In total, 144 chloroplast genomes were used for comparative analysis. The final dataset included 25 chloroplast genomes from this study, as well as 119 from published data (Jain et al., 2022; Xia et al., 2022; Zeng et al., 2022; Dai et al., 2023). The dataset spanned various regions, including East Asia (China, n = 85; Japan, n = 3), Southeast Asia (Vietnam, n = 5; Thailand, n = 3; Cambodia, n = 1), South Asia (India, n = 11), West Asia (Azerbaijan, n = 1), Central Asia (Samarkand, n = 25), North America (USA, n = 1; Bermuda, n = 1), Europe (Spain, n = 1) (**Figure 2**; **Supplementary Table 1**).

### 3.2 Genetic analysis results of mulberries in Samarkand

The PCA analysis results showed that the first two principal components explained 21.91% and 7.39% of the variation, respectively. Although the analysis identified three distinct clusters, this interpretation is limited by the first two PCs (**Figure 1**). Among the samples from Samarkand, five were found to cluster with *M*. *nigra*, while twenty clustered with *M*. *alba*, suggesting that these samples are composed of *M*. *alba* and *M*. *nigra* (**Figure 1**). In line with traditional classification as well as the findings of Zeng et al. (2022), PCA analysis yielded distinct clusters for *M*. *alba* var. *alba* and *M*. *alba* var. *multicaulis* (**Figure 1**). Of the twenty Samarkand samples associated with *M*. *alba*, six clustered with *M*. *alba* var. *alba*, and fourteen with *M*. *alba* var. *multicaulis* (**Figure 1**). The median-joining network analysis confirmed the clustering patterns (**Figure 1**). Pairwise genetic distances were calculated using the same datasets as those used in the phylogenetic analysis. The results revealed that the Samarkand samples were genetically closest to *M*. *alba* var. *multicaulis* (n = 14), followed by *M*. *alba* var. *alba* (n = 6) and *M*. *nigra* (n = 5) (**Supplementary Figure 1**).

The maximum likelihood and the Bayesian trees yielded consistent topologies. Again, the Samarkand samples were distributed across three branches, namely *M*. *alba* var. *multicaulis* (n = 14), *M*. *alba* var. *alba* (n = 6), and *M*. *nigra* (n = 5) (**Figure 1**; **Supplementary Figures 2**, **3**). Notably, other species, such as *M*. *rubra* (n = 1), *M*. *liboensis* (n = 1), and *M*. *cathayana* (n = 2), clustered with *M*. *alba* var. *alba*, while *M*. *macroura* (n = 1), *M*. *australis* (n = 3), and *M*. *indica* (n = 5) clustered with *M*. *alba* var. *multicaulis.* Additionally, *M*. *serrata* (n = 1/1) were found to cluster separately with either *M*. *alba* var. *alba* or *M*. *alba* var. *multicaulis* (**Supplementary Figures 2**, **3**).

## 4 Discussion

In ancient times, the Silk Road represented an expansive network linking East and West, where Central Asia served as a vital hub (Barisitz, 2017). Sericulture was the backbone of the silk industry and a key driver of economic and cultural integration along the Silk Road. Mulberry has historically been an important plant in Central Asia (Mir-Makhamad et al., 2023). However, it is not native to this region. Uzbekistan was among the earliest regions to engage in sericulture (Gu and Pang, 1999). Genetic studies have traced the origins of *M*. *alba* and *M*. *multicaulis* in Europe and the Americas to central or northern China (Dai et al., 2023). Historical and archaeological evidence suggests that sericulture reached Xinjiang by the 3rd century AD from the Hexi Corridor and was introduced to Central Asia during the 3rd to 4th centuries (Gu and Pang, 1999; Zhou and Chen, 2017; Nasirillaev et al., 2023). By the 4th or 5th century, sericulture likely expanded westward via Persia (Zhou and Chen, 2017). Despite these indications, the origins of mulberries in Central Asia remain unclear due to limited direct evidence. Chloroplast genomes, which are highly conserved, maternally inherited, and non-recombinant, offer valuable insights into these origins (Zhang et al., 2023).

Our study found that the 25 mulberry samples from Samarkand, Uzbekistan, could be divided into three distinct genetic groups: The five *M*. *nigra* samples from Samarkand formed a distinct cluster with *M*. *nigra* samples from Xinjiang, China (**Figure 1C**; **Supplementary Figures 2**, **3**). The *M*. *alba* cluster was subdivided into *M*. *alba* var. *alba* (n = 6) and *M*. *alba* var. *multicaulis* (n = 14) (**Figure 1a**). Phylogenetic analysis supported this classification revealing close relationships between these Samarkand samples and those from East Asia, particularly China. Genetic distance analyses corroborated these findings (**Supplementary Figure 1**). Several species, including *M*. *cathayana*, *M. australis*, and *M*. *macroura*, have been classified as variants of *M*. *alba* (Zeng et al., 2015). Additionally, *M*. *indica* is considered a variety of *M*. *alba* (Yang et al., 2023). Genomic studies have consistently grouped cultivated mulberries, such as *M*. *multicaulis* and *M*. *bombycis*, within *M*. *alba* (Jiao et al., 2020; Dai et al., 2023). Our study shows that *M*. *serrata* also nests within the *M*. *alba* clade, which consistent with recent findings (Zeng et al., 2022). Given the phylogenetic relationships (**Figure 1**; **Supplementary Figures 2**, **3**) and prior taxonomic findings (Zeng et al., 2022; Dai et al., 2023), all subsequently mentioned *M*. *alba* are intended to refer to *M*. *alba* var. *alba* and *M*. *alba* var. *multicaulis*. *M. alba* is widely recognized to be native to China (Sharma et al., 2000). The significant genetic connection between the *M*. *alba* from Samarkand and northern China suggests that the *M*. *alba* plants in Central Asia likely originated from northern China (**Figure 1**). Moreover, selective pressure (Ka/Ks) is widely adopted to differentiate neutral mutation (Ka/Ks ≈ 1) from negative (purifying) selection (Ka/Ks < 1) and positive (adaptive) selection (Ka/Ks > 1) (Zhang, 2022). The lower genetic diversity and higher selective pressure observed in the Samarkand samples, compared to the Chinese *M*. *alba* samples (**Supplementary Table 2**), indicate that the Samarkand population has undergone long-term natural or artificial selection, revealing signatures of environmental adaptation (Jiao et al., 2020). Collectively, these findings support the hypothesis of an East Asian origin for Central Asian *M*. *alba*. Conversely, *M*. *nigra* is native to Western Asia and the Caucasus, and was introduced to southern Xinjiang in the 16th century (Zeng et al., 2013). The unique genetic connection between *M*. *nigra* from Xinjiang (China) and those in Samarkand indicates that the *M*. *nigra* likely spread from the Caucasus region through Central Asia to East Asia (**Figure 1**; **Supplementary Figures 2**, **3**). These results point to a dual dissemination pathway, with *M. alba* following an east-to-west route and *M. nigra* tracing a west-to-east migration.

In addition to disseminating mulberry trees, the Silk Road facilitated cultural exchanges related to mulberry cultivation. Ancient mulberry remains from the Niya site suggest that Chinese methods of mulberry cultivation may have influenced Central Asia, with Uzbekistan’s practices resembling these techniques (Nunome, 1979; Zhou and Chen, 2017). The production of mulberry paper, a significant innovation in China, also spread to Uzbekistan via the Silk Road (Helman-Ważny, 2021). The two main species, *M. nigra* and *M. alba*, served different purposes along the Silk Road. *M. alba*, native to northern China and primarily used as silkworm feed, significantly enhanced the quality of silk production in Central Asia. The spread of *M. alba* likely improved the economic output of sericulture in the region. However, *M. nigra*, originating from West Asia and valued for its sweet, juicy fruit, was not suitable for silkworm cultivation (Tutin et al., 1993). While trade and population migration played key roles in mulberry dissemination, climate change may have also influenced the adaptability and growth of mulberries in Central Asia. Mulberry thrives in warm and humid climates, whereas Central Asia’s continental climate is predominantly arid. However, fossil pollen and speleothem δ^18^O records suggest that Central Asia experienced a more humid climate before the 5th to 6th centuries (Cai et al., 2017; Ding et al., 2023), creating favorable conditions for the early establishment of mulberry trees in the region. The subsequent shift to arid conditions, which imposed strong natural selection pressures (Whitney et al., 2019), could be a contributing factor to the low genetic diversity of mulberry in Central Asia (**Supplementary Table 2**) (Whitney et al., 2019; Santana et al., 2025).

In summary, the spread of mulberries in Central Asia is deeply intertwined with the history and influence of the Silk Road, reflecting the region’s role as a conduit for cultural, economic, and agricultural exchange.

## 5 Conclusion

The dissemination and cultivation of mulberries in Central Asia reflect the critical role of the Silk Road in fostering agricultural and cultural exchange. Genetic and historical evidence reveal that the white mulberry (*M*. *alba*), domesticated in northern China, and the black mulberry (*M*. *nigra*), native to West Asia, reached Central Asia through bidirectional exchanges along this ancient trade network. Although Chloroplast genome data provide crucial insights into the genetic origins and dispersal patterns of mulberries in Central Asia, discordant patterns in species relationships between chloroplast genome and whole genome phylogenies can be observed. Our study, as a preliminary investigation focused on a specific transect in Samarkand, highlights the necessity for broader sampling combined with more precise dating of the samples. Accordingly, to further elucidate the details of their origins and dispersal history, it is essential to collect additional samples from across Central Asia and surrounding regions and to conduct whole-genome studies.

## Data Availability

The data reported in this paper have been deposited in the GenBase in National Genomics Data Center, Beijing Institute of Genomics, Chinese Academy of Sciences/China National Center for Bioinformation, under accession number C_AA113447.1 to C_AA113471.1.

## Supplementary material

Supplementary Figure 1 Pairwise genetic distance between the 25 samples in this study and *Morus* spp. (*M*. *nigra*, *M*. *alba* var. *alba*, and *M*. *alba* var. *multicaulis*) based on *p-distance* model.

Supplementary Figure 2 Phylogenetic relationships among *Morus* species based on their chloroplast genomes, with 141 *Morus* accessions and three outgroups (*Ficus*, *Broussonetia*, and *Morus*). The maximum-likelihood tree was constructed with RAxML and the bootstrap value of the four major nodes is 100. Posterior values are marked by dots ranging from red to green, reflecting support values from 1 to 0. Samples from Samarkand are shown in red.

Supplementary Figure 3 Phylogenetic relationships among *Morus* species based on their chloroplast genomes, with 141 *Morus* accessions and three outgroups (*Ficus*, *Broussonetia*, and *Morus*). The Bayesian tree was constructed with Beast and the posterior value of the four major nodes is 1. Posterior values are marked by dots ranging from red to green, reflecting support values from 1 to 0. Samples from Samarkand are shown in red.
