# Supplementary figures and images for: The chloroplast genome elucidates the origin of mulberry in Central Asia
Source: Front Plant Sci. 2025 Aug 28;16:1592308. doi: 10.3389/fpls.2025.1592308 (PMC12423069; doi:10.3389/fpls.2025.1592308)

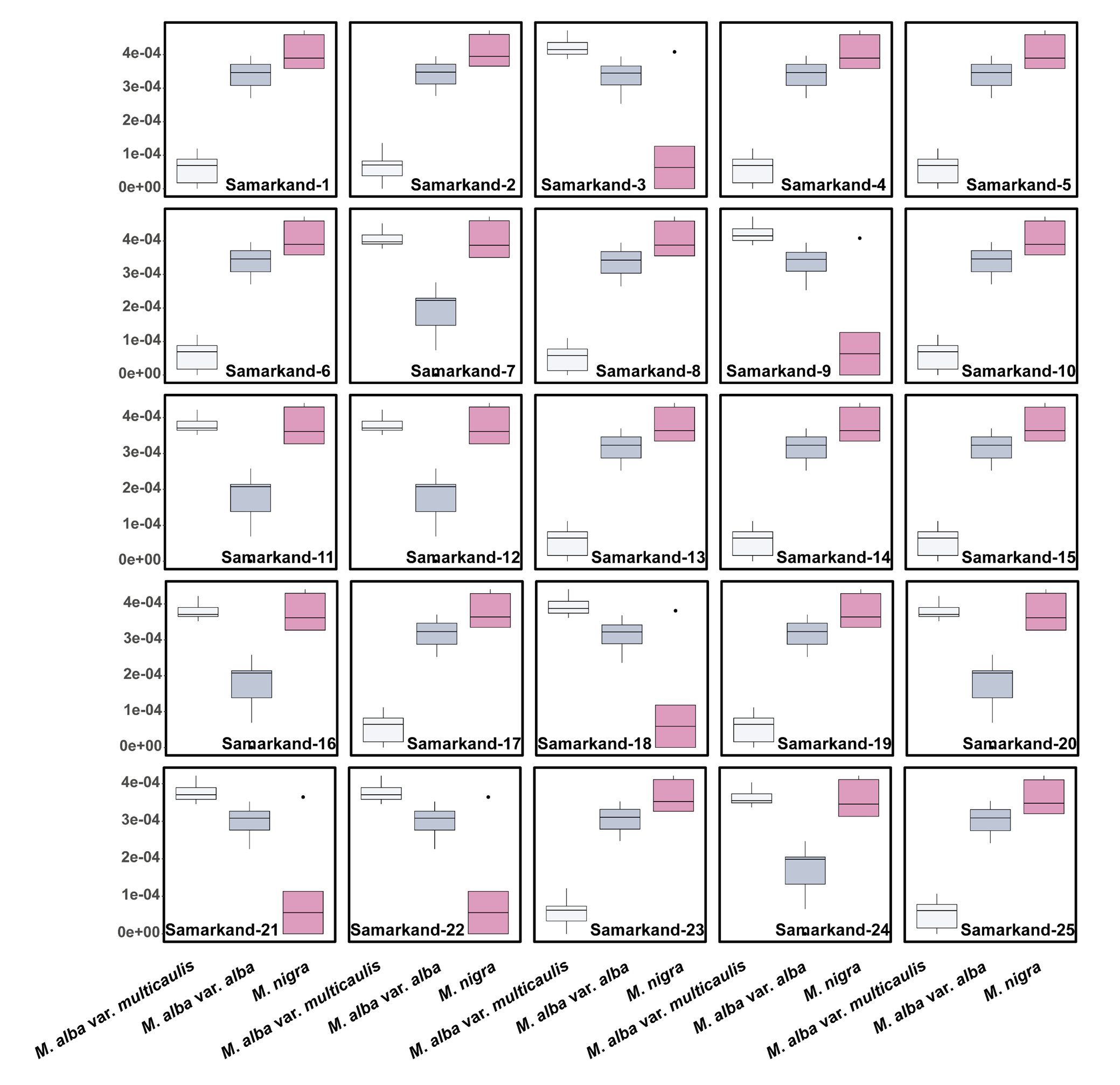

Supplement: Supplementary Figure 1 — Pairwise genetic distance between the 25 samples in this study and Morus spp. (M. nigra, M. alba var. alba, and M. alba var. multicaulis) based on p-distance model. [file Image1.tif]

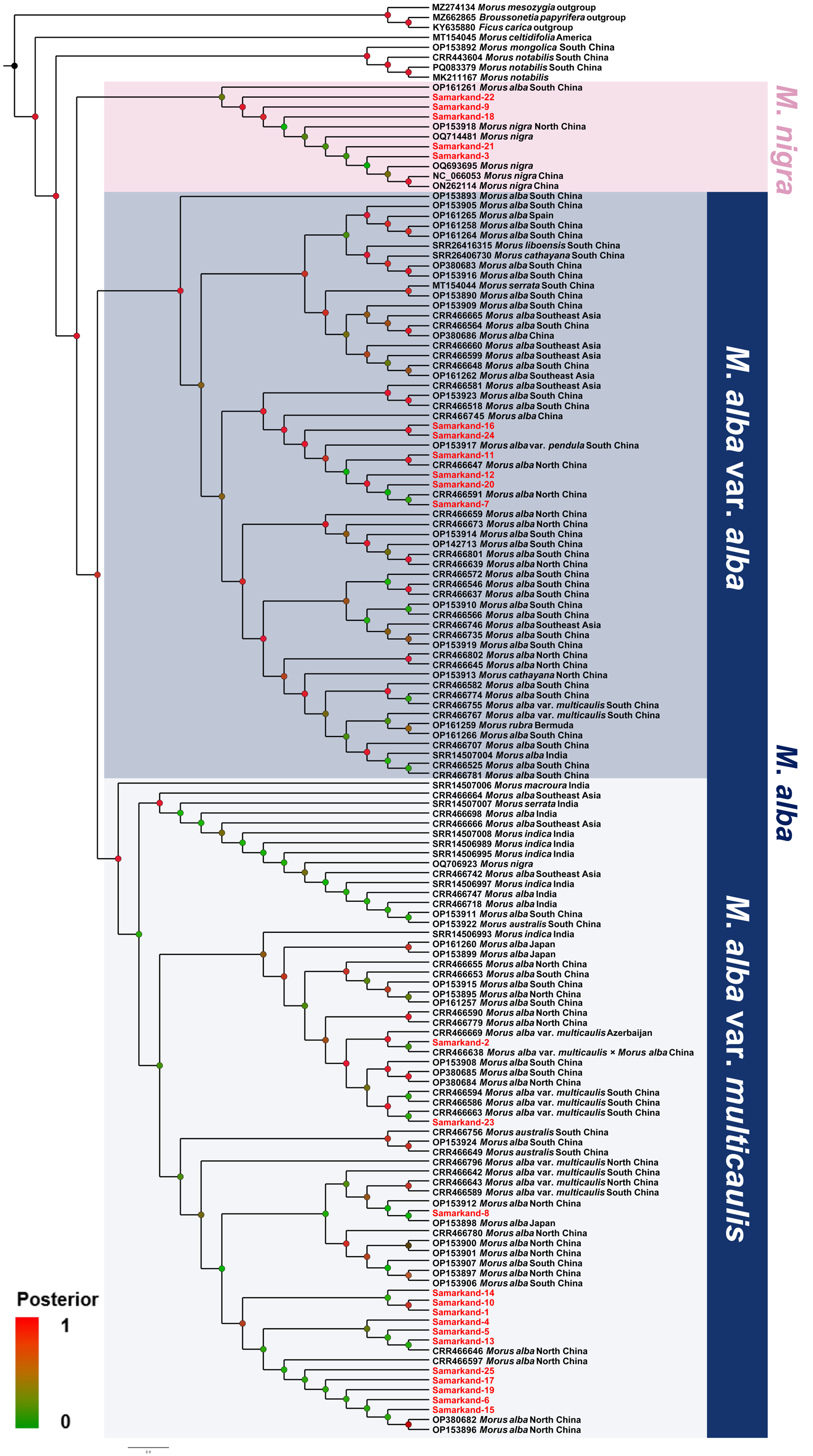

Supplement: Supplementary Figure 2 — Phylogenetic relationships among Morus species based on their chloroplast genomes, with 141 Morus accessions and three outgroups (Ficus, Broussonetia, and Morus). The maximum-likelihood tree was constructed with RAxML and the bootstrap value of the four major nodes is 100. Posterior values are marked by dots ranging from red to green, reflecting support values from 1 to 0. Samples from Samarkand are shown in red. [file Image2.tif]

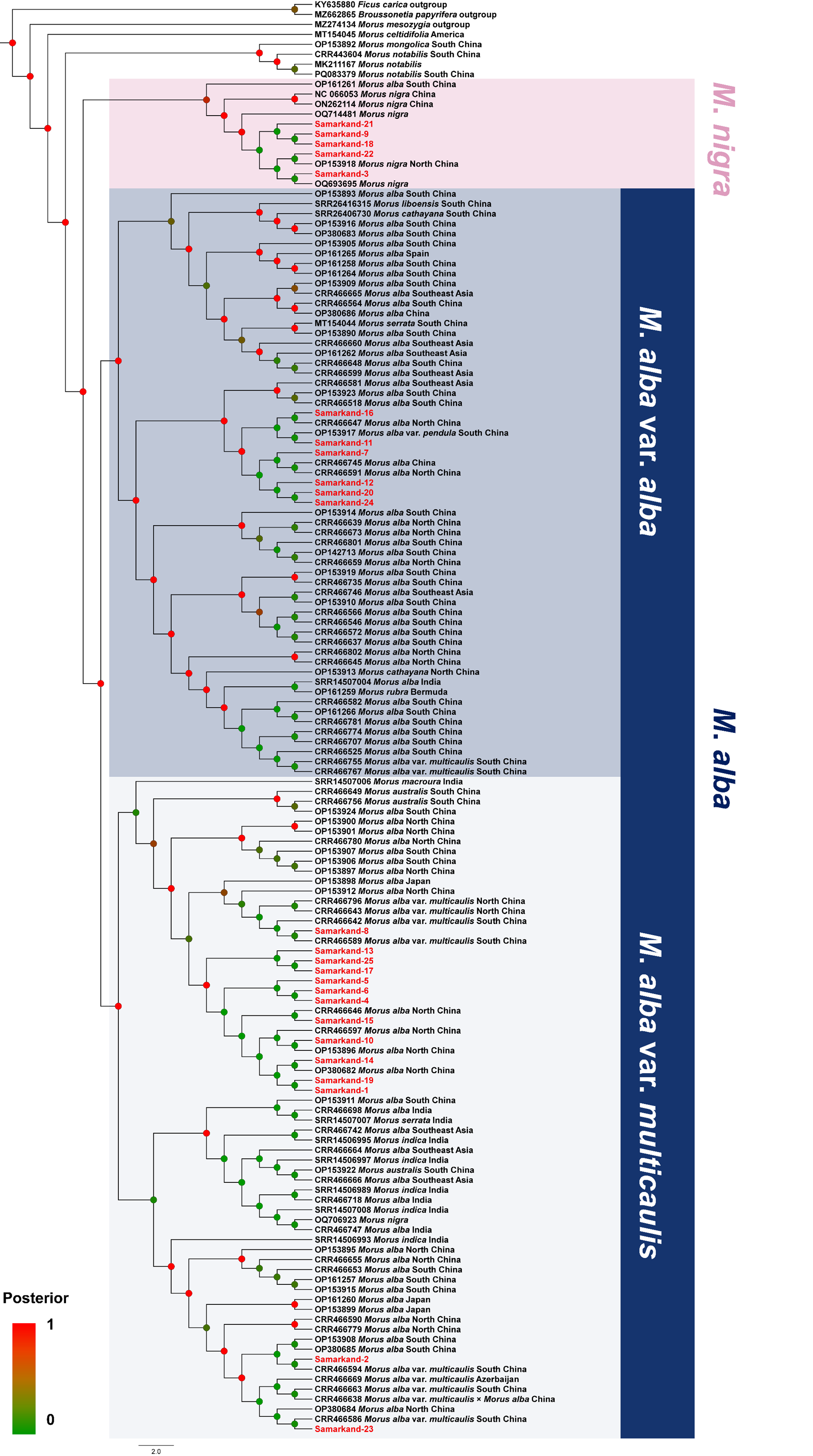

Supplement: Supplementary Figure 3 — Phylogenetic relationships among Morus species based on their chloroplast genomes, with 141 Morus accessions and three outgroups (Ficus, Broussonetia, and Morus). The Bayesian tree was constructed with Beast and the posterior value of the four major nodes is 1. Posterior values are marked by dots ranging from red to green, reflecting support values from 1 to 0. Samples from Samarkand are shown in red. [file Image3.tif]
